# Revisiting Vision Rehabilitation

**DOI:** 10.3389/fnsys.2017.00082

**Published:** 2017-11-01

**Authors:** Claire Meyniel, Bahram Bodaghi, Pierre-Yves Robert

**Affiliations:** ^1^Department of Neurophysiology, Pitié-Salpêtrière Hospital, Paris, France; ^2^Department of Ophthalmology, University of Pierre et Marie Curie, Paris-Sorbonne University, Paris, France; ^3^Department of Ophthalmology, Pitié-Salpêtrière Hospital, Paris, France; ^4^Department of Ophthalmology, Limoges Hospital, Limoges, France; ^5^Department of Ophthalmology, University of Limoges, Limoges, France

**Keywords:** low vision, rehabilitation, visual impairment, blindness, quality of life, recovery, restoration, vision loss

## Abstract

Low vision is a condition caused by eye or brain disease, in which visual acuity is 20/70 (3/10 or 6/18) or poorer in the better-seeing eye and cannot be corrected or improved with regular eyeglasses. It impacts personal ability to perform vision-dependent tasks as activities of daily living, walking, reading or using a computer. Rehabilitation is a multidisciplinary training dedicated to improve patients’ functional abilities and quality of life. It has to be personalized to every individual situation, whatever the underlying pathology.

Low vision refers to conditions of reduced vision uncorrectable by glasses, medication or surgery. Two hundred eighty five million people are estimated visually impaired worldwide. Apart curable etiologies such as cataract and refractive errors, main causes of low vision are glaucoma, age related macular degeneration, corneal opacities and diabetic retinopathy. Visual impairment affects mainly the elderly with a proportion over 80% among the 60-year old and more (Bourne et al., 2013).

Low vision impacts personal abilities to perform vision-dependent tasks for many activities of daily living, including reading, cooking or matching clothes. The consequences of visual impairment on daily life may widely vary. Patients with central scotoma will experience troubles for reading and performing activities requiring near vision. Associated peripheral field loss will rather cause difficulties to detect obstacles during walking. Additionally, dark adaptation disorders may affect ability to see at night with subsequent negative impact on gait.

Therefore low vision rehabilitation, as a challenge to improve functional ability and other general aspects, needs to be adapted to every patient situation. Rehabilitation delivers a multidisciplinary training including visual strategies, occupational therapy, mobility and adaptation of optic and non optic aids (Dagnelie, 2013). This article reflects the state-of-the-art in visual rehabilitation, based on a comprehensive review of center practices as described in published literature.

## Classification

Assessment of exact visual impairment is one of the main conditions for a successful rehabilitation. Low vision relies on visual acuity and visual field, which may be independent contributing factors.

The World Health Organization (WHO) provides a specific meaning for low vision as follows: “A person with low vision is one who has impairment of visual functioning even after treatment and/or standard refractive correction, and has a visual acuity of less than 6/18 to light perception, or a visual field of less than 10 degree from the point of fixation, but who uses, or is potentially able to use, vision for planning and/or execution of a task” (World Health Organization, 1980; ICO, 2002).

Visual acuity impairment is defined as a visual acuity lower than 20/70 (Snellen notation) or 3/10 (decimal notation) or 6/18 (6 m notation) in the better seeing-eye even after treatment and/or standard refractive correction. The WHO classified visual impairment in five different categories: 1: moderate visual impairment, 2: severe visual impairment, 3–5: blindness (3: profound blindness, 4: near total blindness and 5: total blindness; Table 1).

**Table 1:** Definition of low vision relying on visual acuity and visual field (World Health Organization, 1980; ICO, 2002).

Range of vision loss	Visual acuity (VA)	Reading ability (statistical estimates)	Visual field (VF) radius (diameter)	Orientation and mobility ability (statistical estimates)
**Moderate visual impairment**	3/10 > VA > 1/10^(1)^ or 20/70 > VA > 20/200^(2)^ or 6/18 > VA > 6/60^(3)^	**Normal with aids** - Strong reading glasses - Moderate power magnifiers	20° > VF > 10° (40° > VF > 20°)	**Normal with aids** - Scan for obstacles
**Severe visual impairment**	1/10 > VA > 1/20^(1)^ or 20/200 > VA > 20/400^(2)^ or 6/60 > VA > 3/60^(3)^	**Restricted with aids** - High power magnifiers	10° > VF > 5° (20° > VF > 10°)	**Restricted with aids** - Continuous scanning with cane to detect obstacles - Vision as adjunction for identification
**Blindness**	1/20 > VA^(1)^ or 20/400 > VA^(2)^ Or 3/60 > VA^(3)^	**No visual reading** - Vision substitution skills (braille, talking books, audio production equipment)	5° > VF (10° > VF)	**Visual orientation unreliable** - Continuous scanning with long cane to detect obstacles - Hearing - Guide dog

*^(1)^Decimal notation. ^(2)^Snellen notation. ^(3)^6 m notation*.

Visual field is also taken into account for categorizing the patients. A patient with visual field of the better eye of no greater than 10° in radius around central fixation places the patient in category 3 or higher (World Health Organization, 1980; Table 1).

## Rationale for Treatment

Apart its direct functional impact, visual impairment increases the risk of injury or miscellaneous disorders. Furthermore, when vision loss coexists with other health problems, such as hearing loss or cognitive deficiency, the resulting consequences are usually higher than the sum of the two impairments. For example, increased risk to be involved in a car accident, to fall, to undergo bone fracture, to be unemployed, to be socially isolated or to make errors in drug self-administration and to develop depression or cognitive disorders.

- Fall: Visually impaired individuals have an increased risk of more than twice to fall and four times to sustain a hip fracture (Klein et al., 1998; Shen et al., 2014). Factors identified to prevent falls are exercises, physical therapy and vitamin D supplementation (Michael et al., 2010).
- Depression: Several studies following patients with low vision have reported that over 30% develop depression and over 15% anxious disorders. Problem solving treatments associated with referral to patient’s physician have shown improvement of the depressive symptoms (van der Aa et al., 2015; Nollett et al., 2016).
- Cognitive disorders: Even though cognitive and visual impairments are more frequent in elderly people and thus may coexist, a causal relationship between the two conditions is still uncertain. Several studies have reported increased cognitive impairment in age related macular degeneration and in visually impaired patients hospitalized in geriatric units (Pham et al., 2006; Woo et al., 2012; Fukuoka et al., 2015). In England, a large cohort study did not report significant association between age related macular degeneration and dementia (Keenan et al., 2014).
- As a high proportion of patients with vision loss lose their jobs, associated factors of unemployment are identified, such as diabetic pathology, being a woman and young age at the diagnosis (lower than 55 years old). Only 24% of women with low vision are working vs. 58% of men (Sherrod et al., 2014).

## Eye Care Providers

Ophthalmogists subspecialized in low vision assess residual visual function with visual acuity, refraction, contrast sensitivity, visual field, reading speed and reading distance. They also detect improvable abnormalities as cataract or macular cystoid edema that can modify vision evolution. Measurement of contrast sensitivity helps to analyze functional vision. Patients with impairment of low contrast perception may have trouble for mobility and patients with impairment of high contrast perception may have more difficulties to perform near vision tasks such as reading and daily living activities. Such patients might be improved by increasing illumination and video magnifier to rise or reverse the contrast (Latham and Tabrett, 2012). Visual field analysis includes characterization of fixation and search for scotomas and peripheral defects. It is performed first eye by eye and after both eyes together. Oculomotor functions, head turns, deviated gaze need to be detailed. Microperimetry is a valuable machine to improve fixation stability, reading speed and visual acuity in studies following patients with geographic atrophy (Ramírez Estudillo et al., 2017).

Patient’s ability to perform visual tasks and potential benefits of rehabilitation are estimated. Influencing factors as hearing loss, cognitive troubles, tremor, sensory or motor deficit and depressive syndrome need to be identified. Presence of functional symptoms as hallucinations should be also asked systematically as most of the time hallucinations are related to the Charles Bonnet’s syndrome. In those cases, medical doctors can reinsure patient and discuss interest of medical treatment despite limited efficiency (Crumbliss et al., 2008; Schadlu et al., 2009; Grüter et al., 2016).

At the end of the evaluation, a specific program of rehabilitation is proposed to the patient and adapted to his/her functional vision and his/her abilities, taking into account employment, hobbies, family and living situation, independently of the underlying pathology.

The training involves visual rehabilitation officers. They are trained professionals after at least 3 years of degree qualification: optometrists, orthoptists, orientation and mobility instructors, occupational therapists, physiotherapists and psychologists and sometimes other professionals (Table 2). They work with patients at home or in rehabilitation centers with adapted rooms, such as kitchens, bathrooms or dressings. From one to three times a week, according to patient’s needs, visual rehabilitation officers train the patients to learning new strategies and to improve their quality of life (Markowitz, 2006). Repartition of this multidisciplinary work varies among countries.

**Table 2:** Multidisciplinary training of visual rehabilitation: the work force.

Workforce	Rehabilitation	Equipment
**Medical doctor**	Assessment of causes of vision loss, visual functions and functional vision	Eye examination including high and low contrast distance, near visual acuity and refraction
		Awareness and education of people with low vision or blindness and the community
**Optometrists**	- Visual skills rehabilitation training as eccentric fixation or visual scanning - Strategies to improve reading - Use of optical devices	Spectacle magnifiers to 20D and spectacle telescopic devices (2× and 4×…) Foldable and hand-held magnifiers with and without built-in light source (5D– 17D…) Stand magnifiers (12D– 50D…) Dome and bar magnifiers Monocular hand held telescopes (4×, 6×, 8×…) Filters with shades of luminous transmission
**Orientation and mobility instructors**	- Walking - Guide training - outside trip - use of public transports	Mobility devices including white cane and digital devices
**Daily living activities instructors**	- Participating in activities in daily living: meal preparation, personal care, cleaning, money issue	Non-optical devices such as reading stand, lamps and good contrast items
**Communication instructors**	- Braille - Modifications to computer software - Hand writing	Braille writing and reading equipment, digital technology Tactual writing devices Computer, Audio production equipment and devices
**Social workers**	- Entitlements	Disabled parking badge Personal taxes allowance Founding for optical and non optical devices
**Counsellors or psychologists**	- Evaluation of patient concerns - Detection of anxiety and depressive symptoms	Family interactions Support groups Self-management programs

## Procedures

### Reading

When possible, reading is the first task required during rehabilitation. Fluent reading requires a minimal visual acuity of 20/50, a minimal visual field of 2° to the right and the left as well as holding position of 250 ms between saccades (Legge et al., 1997).

The patients with a central scotoma are trained to use an eccentric fixation in an intact area at the margin of the scotoma called preferred retinal locus. Theoretically, the most favorable location is positioned in the upper visual field. Some patients preferred a fixation such as areas located on the right or the left retina of the scotoma (Trauzettel-Klosinski, 2010).

In a second step, the helpfulness of a magnifier is tested, looking for the smallest character size that can be read fluently. A large panel of magnifiers exists. For example: spectacle magnifiers, foldable and hand-held magnifiers with and without built-in light source, stand magnifiers, dome and bar magnifiers. Furthermore, telescopes as monocular hand held telescopes, spectacle telescopic devices can be used for long distance viewing. Improvement of reading abilities higher than 90% was reported after magnifying visual aids for patients with central scotoma related to age-related macular degeneration (Nguyen et al., 2009). Sufficient illumination without glare is also important with the adaptation of light. In case of photophobia, modification of the brightness can be proposed as well as filters with different shades of luminous transmission. Filters can be included directly on the glasses with the optimal refraction. Removable filters or large covering glasses are other options (Virgili et al., 2013).

Patients with specific visual field defects can also improve their reading abilities:

- In case of constricted visual field, as in retinal dystrophy or late glaucoma, the central seeing island might be too small for reading. Contrast enhancement and reduction of the size of the text can be proposed.
- Patient with ring scotoma may have discordance between impaired reading performances and good visual acuities in case the central island is too small. Training to an eccentric fixation outside of the ring scotoma can help for reading.
- Homonymous hemianopias generate variable difficulties for reading regarding the side of the deficit. Patients with a left homonymous hemianopia have troubles to find the next line of the text. Using a guide as a ruler or a finger helps the eyes to come back at the beginning of same line and then to step to the beginning of the next line. In case of right homonymous hemianopia, difficulties of reading are the consequences of the impossibility to anticipate the next letters. An eccentric fixation that expends the perception in the right field may help to regain fluent reading. Additionally, a saccadic training to explore the hemianopic side can be proposed (Trauzettel-Klosinski, 2010).

### Activities of Daily Living

Improving autonomy in daily living activities is the second most frequent demand of visually impaired patients. The main domains are cooking, personal care, gesture recognition and financial management. Regarding the patient’s needs, rehabilitation can also include clock reading, self-administration medication, shopping, cleaning, ironing, sewing, knitting or how to make-up. Non optical aids are proposed as vocal clock, lamp or contrasted items (Finger et al., 2014).

### Communication

Communication for adults with an acquired visual deficiency is nowadays based more on computer or cell phone than on Braille. Braille is a tactile reading system coding for text within two different forms: Alphabetic Braille writes out each letter and Literary Braille is a contracted form of writing. As Alphabetic Braille is mainly used for labeling items and writing short messages, Literary Braille is preferred to read longer texts such as books (Jiménez et al., 2009). Using a computer is possible even for patients with severe vision loss or blindness. Large print keyboards, magnification softwares, typing, audio-screen readers or text to speech are among the possibilities to help visually disabled to use a computer. Pads are another option for a simple access to data processing (Mednick et al., 2017). Cell phones may be adapted to patient needs, from an emergency alarm to internet access with audio-screens readers. Tactile thermoformed guide for letters and bank checks can also improve hand-writing.

### Mobility

Generally driving requirements are not fulfilled by patients with low vision. Patient’s safety for walking is evaluated in a protected area. Work begins with landmark catches, improvement of the posture, balance and footstep. Long canes or white canes may be proposed if necessary but requires training before usefully use them for scanning. Training includes street crossing, using street noises for example or public transport autonomy if needed (Virgili and Rubin, 2010). Guide dog training, limited to patients with severe visual loss or blindness, required the prior acquisition of autonomy for an independent ambulation (Refson et al., 1999).

## Effectiveness

Many studies have reported the effectiveness of rehabilitation and have evidenced the improvement of reading ability with optical devices and low vision aids (Virgili et al., 2013). In contrast, home visit of a vision specialist to adapt optical devices and the use of prism spectacles for reading have not shown any additional benefit yet (Binns et al., 2012).

In a prospective study including 779 patients, Goldstein found that half of the patients had a clinically meaningful difference in overall visual ability after rehabilitation. Regarding the functional domain, 44% of the patients reported an improvement for reading, 38% for visual motor function, 33% for visual motor processing and 27% for mobility (Goldstein et al., 2015). Furthermore, the severity of depressive symptoms decreased after rehabilitation. The effectiveness of this multidisciplinary training is however difficult to evaluate. Only modest improvements of vision related quality-of-life were reported, the progress was observed mainly on items related to near vision (Binns et al., 2012; Goldstein et al., 2015).

## Innovations and Perspectives

The development of new technologies begins to change visual rehabilitation processes.

Electronic canes help patients to detect nearby objects. This technology consists in a small device attached to a standard white cane. Ultrasounds sensors detect obstacles ahead using ultrasonic waves bouncing back (Pallejà et al., 2010). More recently, optical character recognition devices convert visual information such as text, monetary denominations and faces into spoken words. The device includes a miniature camera clipped onto the wearer’s eyeglass frame. In a prospective study including 12 patients, the use of such a device increased scores of daily living activity scale (Moisseiev and Mannis, 2016).

Several studies reported the interest of repetitive transcranial stimulations, as non-invasive methods for restoring vision (Halko et al., 2013). Transcranial magnetic stimulation generates focal and transitory cortical changes; this technique is mainly used to explore the cortical visual system with virtual lesions approach (Halko et al., 2013). Transcranial direct current stimulation is another technic to induce changes of cortical excitability. Repeated in daily sessions, it may modulate brain network plasticity. After unilateral occipital strokes, cortical transcranial direct current stimulation combined with rehabilitation enhanced recovery of homonymous hemianopic visual field defects (Gall et al., 2015; Matteo et al., 2016).

Retinal implants appear to increase vision with acceptable safety profiles. Implanted patients report significant improvement of quality of life, even though the amelioration of functional vision generated by retinal device remains limited (Humayun et al., 2016). Artificial vision has already shown an efficacy in patients suffering from retinal photoreceptors degeneration (Mills et al., 2017). After the surgery, rehabilitation has a major place to maximize the benefits of the technology as adjustments to light and contrast conditions, objects recognition and surroundings localization (Dagnelie, 2012).

## Conclusion

As a direct consequence of aging population worldwide, a marked increase in the prevalence of low vision is expected. Even though the developments of low vision concern, many patients are still not referred to rehabilitation care. Education of eye care professionals and expansion of rehabilitation centers could be options to reduce the burden of low vision worldwide.

## Author Contributions

CM conceived and drafted the manuscript and is guarantor for the content. BB and P-YR edited the manuscript.

## Conflict of Interest Statement

The authors declare that the research was conducted in the absence of any commercial or financial relationships that could be construed as a potential conflict of interest.
